# A Novel Wearable Sensor for Measuring Respiration Continuously and in Real Time

**DOI:** 10.3390/s24206513

**Published:** 2024-10-10

**Authors:** Amjad Ali, Yang Wei, Yomna Elsaboni, Jack Tyson, Harry Akerman, Alexander I. R. Jackson, Rod Lane, Daniel Spencer, Neil M. White

**Affiliations:** 1 Smart Wearable Research Group, School of Science and Technology, Nottingham Trent University, Nottingham NG11 8NS, UK; amjad.ali@ntu.ac.uk (A.A.);; 2 School of Electronics & Computer Science, University of Southampton, Southampton SO17 1BJ, UKnmw@ecs.soton.ac.uk (N.M.W.); 3 Clinical Care, University Hospital Southampton NHS Foundation Trust, Southampton SO16 6YD, UK; 4 Perioperative and Critical Care Theme, NIHR Southampton Biomedical Research Centre, University Hospital Southampton NHS Foundation Trust, Southampton SO16 6YD, UK; 5 Integrative Physiology and Critical Illness Group, Clinical and Experimental Sciences, Faculty of Medicine, University of Southampton, Southampton SO16 6YD, UK; 6 Zelemiq Ltd., Salisbury SP5 1EZ, UK

**Keywords:** electronic textiles, wearable sensors, respiratory sensors

## Abstract

In this work, a flexible textile-based capacitive respiratory sensor, based on a capacitive sensor structure, that does not require direct skin contact is designed, optimised, and evaluated using both computational modelling and empirical measurements. In the computational study, the geometry of the sensor was examined. This analysis involved observing the capacitance and frequency variations using a cylindrical model that mimicked the human body. Four designs were selected which were then manufactured by screen printing multiple functional layers on top of a polyester/cotton fabric. The printed sensors were characterised to detect the performance against phantoms and impacts from artefacts, normally present whilst wearing the device. A sensor that has an electrode ratio of 1:3:1 (sensor, reflector, and ground) was shown to be the most sensitive design, as it exhibits the highest sensitivity of 6.2% frequency change when exposed to phantoms. To ensure the replicability of the sensors, several batches of identical sensors were developed and tested using the same physical parameters, which resulted in the same percentage frequency change. The sensor was further tested on volunteers, showing that the sensor measures respiration with 98.68% accuracy compared to manual breath counting.

## 1. Introduction

Common chronic respiratory diseases (CRDs) including asthma, sleep apnoea, and chronic obstructive pulmonary disease currently affect more than 435 million people globally [1,2]. Continuous monitoring of respiratory activity is crucial in detecting or anticipating critical situations that can potentially save lives if detected early on [2]. As a result, there has been a push to develop methods for non-invasively and efficiently measuring human respiratory rate, along with having a medically acceptable standard value for an accuracy of ±2 breaths per minute [3,4]. Waveform capnography is the gold standard for measuring respiratory rate in clinical practice [5,6]. Another similar approach suggests a mouthpiece respiratory rate monitoring sensor [7,8], which consists of overlapping interdigitated pattern electrodes produced by inkjet printing of silver nanoparticles. These electrodes were then covered with graphene, whose conductivity changes with flowing air pressure, temperature, and humidity. However, this kind of sensor needs to be breathed through the mouthpiece and cannot be used continuously in daily life. Additionally, it is necessary to conduct extensive research to determine if there are any potential side effects on the human body if graphene or silver particles are accidentally released from the sensor structure and inhaled by the subject. Mahbub et al. suggested a patch of Polyvinylidene-Fluoride film (piezoelectric transducer), which could monitor chest vibrations by producing a varying 500 mV voltage [9]. However, this type of sensor is highly sensitive to motion artefacts, arm movements, and walking. Respiratory rate sensors have also been manufactured directly on cloths by exploiting conductive textiles, such as textile-based stretchable belts, resistive sensors based on strain sensing, pressure-sensing foam, capacitive sensors, and textile-based resonant sensor tags [10,11,12,13,14,15], for example, in monitoring changes in abdomen circumference using tight belts placed around the rib cage [9,10,11,12]. The tight belt consists of insulated copper wire, which is knitted in tabulated mesh form in a stretchable textile to produce a stretchable belt [11]. The belt is firmly wrapped around the test subject torso and the inductance variation was monitored with torso expansion and contraction. The change in inductance was utilised to track breathing. A compressible foam was developed by Sarah et al. in [13,14], where the resistance decreases or increases with the application of external pressure on the foam. The foam is encapsulated in a non-stretchable textile belt and firmly wrapped around the torso. The expansion and contraction of the torso during breathing are recorded in the form of resistance variations. However, these sensors can be uncomfortable as they need to be tightly positioned. Additionally, the externally applied force, rubbing, and flexing durability on compressible foam-based sensors could lead to uncertainty in respiratory rate monitoring. The strain sensors reported by Atalay et al. [16] and Huang et al. [17] consist of knitted loops of bare conductive fibres with non-conductive textile fibres. In their unstretched form, the bare conductive fibres make contact at various points within the loops, providing a direct (shorter) path for the current flow and reducing resistance due to the shorter path length. As the textile stretches, the contact area between conductive fibre loops decreases, and in some cases, there are no contact points within the loops, causing the current to flow only through the wire path, resulting in increased resistance due to the full length of the wire. However, the tight fit of resistive sensors around the torso can cause discomfort during extended use.

Min et al. in [18] and Hoffmann et al. in [19] reported a capacitive-based respiratory rate sensor. The working mechanism is based on encapsulating a compressible foam between the two capacitive electrodes, which is then encapsulated in a non-stretchable textile belt. During respiration, the chest’s applied force on the compressible foam reduces the gaps between the capacitive electrode, which is used for respiration monitoring. However, these sensors are susceptible to slipping and can lead to inaccurate measurements. These capacitive sensors can be uncomfortable to wear due to the fastened belt, which may cause the body to overheat and sweat. A circular two-electrode-based capacitor is proposed for respiratory rate monitoring [20]. It is based on monitoring the small movement of the thorax with respiration from a distance of 0.2 m. Although this system is designed for monitoring respiratory rate during ECG and can effectively monitor respiration in ideal conditions when there are no nearby objects except the test subject, the sensor capacitance, however, could potentially be affected by nearby metallic and non-metallic objects. Additionally, this proposed sensor, designed to monitor respiratory rate during ECG, has a larger size of 20 cm. Due to the larger size, it is not practical to be used in daily life. A rectangular capacitive-based multimodal sensor was developed on stretchable textiles [21]. It detects the change in capacitor area due to the stretching nature of the capacitor. The work elaborates on how the sensor can monitor abdomen expansion, elbow movements, thorax expansion and compression with breathing, and heart rate by wrapping the capacitive sensor around the abdomen, elbow, thorax, and neck, respectively. Although the sensor could monitor multiple vital signs, the tight wrapping could be uncomfortable for a subject all day long. Additionally, the study missed the immense effect of noises, such as changes in humidity, temperature, vibrations, rubbing, flexing durability, and pressure. The team [22,23] has developed a wearable respiratory sensor for continuous respiratory rate monitoring in patients, both at rest and during exercise [22,23], based on the principle of a capaciflector [24].

In this study, significant efforts were made in the designing of a novel sensor, with the goal of obtaining a flexible, lightweight, and comfortable respiratory sensor integrated within fabric allowing for real-time continuous monitoring. This study establishes the methodology for achieving the optimal design in the presence of artefacts. Based on simulation and empirical results, the most sensitive design was used to capture the breathing rate of a test subject.

## 2. Sensor Design, Simulation, and Manufacturing

### 2.1. Ansys Simulation

The proposed textile-based sensors were designed, simulated, and optimised using ANSYS (Twin Builder 2022 R1), a computational Multiphysics software. The objective is to find optimum sensor electrode ratios that produce higher electric field distribution in the nearby objects. The design consists of three electrodes: the sensor, reflector, and ground. Through simulations, various dimensions of each electrode were studied to determine the optimal structure.

**Figure 1:**
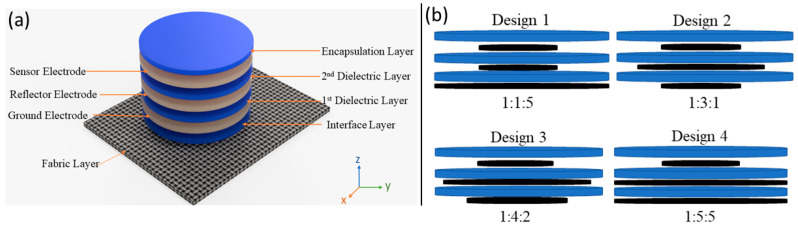
(**a**) The layout of the designed sensor. (**b**) The structure of the four sensor designs and ratio combinations of the sensor, reflector, and ground electrode.

**Figure 2:**
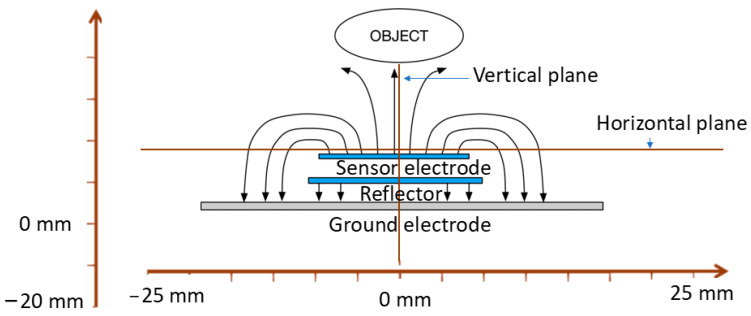
Diagram of simulation methodology conducted to obtain electric field distribution around each sensor design.

**Figure 3:**
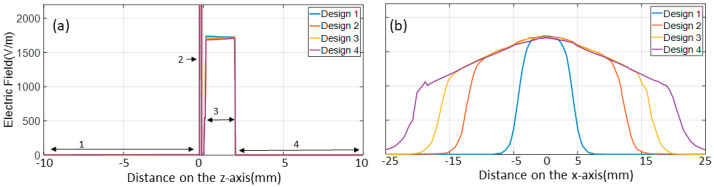
(**a**) The computed vertical electric field is (1) below the ground electrode, (2) between the ground and reflector electrode (has a high electric field which reaches up to 75,000 v/m due to a thin dielectric layer), (3) between the sensor and phantom (design 1: vertical electrical field peak is 50 v/m higher than the rest of the design), and (4) within the phantom (the phantom is a glass cylinder of 80 mm diameter set to a dielectric constant of 5 with a glass wall thickness of 2.5 mm and filled with water having dielectric constant of 80). (**b**) The computed horizontal electric field distribution across the four sensor designs.

The layout shown in Figure 1a was consistent across all designs. The conductive electrodes are modelled with silver and are placed between insulating polyimides. The idea is to operate the sensor along with its electronics with a small lithium 3 V battery (CR2032). Therefore, the ground electrode is assigned a potential of 0 V, while the reflector and sensor electrodes are at the same potential of 3 V. The optimisation process aimed to select the most effective sensor electrode dimensions. According to previous research [23], a reduced capacitance between the sensor and ground electrode (Csg) and a greater capacitance between the sensor electrode and the object (Cso) it is detecting lead to increased sensitivity of the sensor. Figure 1b shows four designs, where each design is different from the others by having different ratio combinations of sensor, reflector, and ground electrode diameters. The sensing mechanism of the proposed sensor consists of capacitance variations between the sensor electrode and the object that comes closer to the sensor electrode, as shown in Figure 2. Figure 2 also demonstrates that the multilayer structure directs the sensor electrode’s electric field toward the object to be sensed. Simultaneously, the sensor electrode’s electric field is shielded by the ground and reflector electrodes from objects approaching from the ground side, preventing interference from that direction. Figure 2 shows the methodology used to obtain the electric field, where vertical and horizontal lines are placed at the centre of the sensor each time. These designs are obtained through an optimisation process that involves adjusting the diameter of the sensor, reflector, and ground electrode to achieve an optimum vertical electric field from the sensor electrode, as shown in Figure 3a.

**Figure 4:**
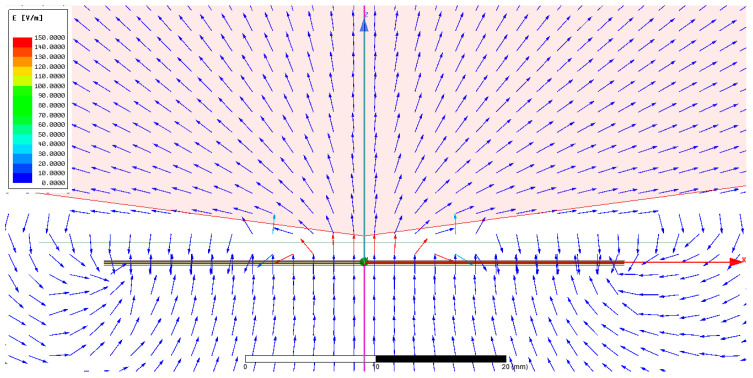
The sensor capacitance was recorded when the phantom was located at z = 1, 5, 10, 15, 20, and 25 mm distances. The electric field distribution of the final sensor designs across the perpendicular plane and the parallel plane. The fringing field effect is present at the corners between the sensor and ground electrodes.

In order to compute different designs’ vertical and horizontal electric fields, the sensor is located in a 3D simulating environment plan at x = 0 mm, y = 0 mm, and z = 1 mm, as shown in the block diagram in Figure 2, while moving the phantom on the z-axis at values such as z = 1, 5, 10, 15, 20, and 25 mm. The analysis of Figure 3b shows that design 4 exhibited a more dispersed electric field distribution within objects placed parallel to the sensor during simulations, with a reduced electric field travelling vertically. In contrast, design 1 demonstrated a more focused electric field in the vertical direction and limited spread in the horizontal directions, resulting in a more directional field toward the object. Furthermore, the aim is to direct the electric fields toward the object as much as possible. Therefore, significant efforts were made by simulating and analysing different ratio combinations of three electrodes. The simulated study suggests that, by setting a ratio of 1:3:1, the electrical field of the sensor electrode is directed toward the object at the maximum and has minimal fringing field effect between the sensor and ground electrode, as shown in Figure 4. To evaluate the sensitivity to changes in object permittivity, three phantoms made of gel, acetone, and water were modelled and positioned in close proximity to the sensor in each design.

**Figure 5:**
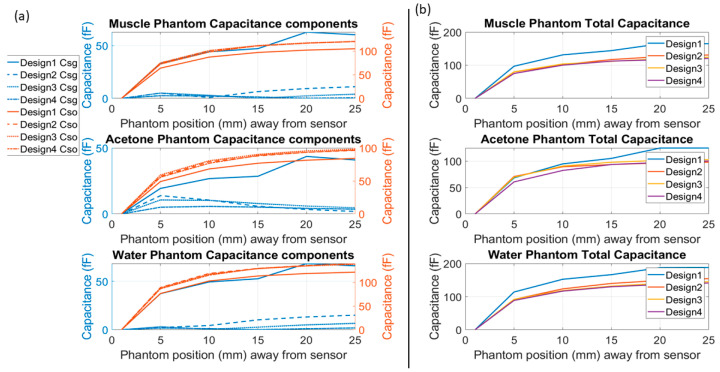
Simulation capacitance results were obtained with 3 phantoms: muscle, acetone, and water. (**a**) shows the capacitance of each design between the sensor and ground electrodes, which is referred to as Csg and given a blue colour along with its scale on the left side of each graph. The capacitance between the sensor and the object is referred to as Cso, which is given an orange colour along with its orange scales on the right side of each graph. (**b**) shows each design’s total capacitance toward the phantom.

The simulation results, illustrated in Figure 5, showcased the capability of the optimised designs to detect the capacitance variations based on the object’s permittivity. It was shown that enhancing capacitance variations between the sensor electrode and the object (Cso) can enhance the sensor’s sensitivity. These findings highlight the importance of optimising the electric field distribution in sensor design, as it directly affects the sensor’s ability to detect and measure capacitance changes with the object in the surrounding environment.

Figure 5a shows the sensor electrode capacitance with the ground electrode as well as with the object (which is the corresponding phantom) for comparison. In contrast, Figure 5b shows the sensor capacitances with the corresponding phantom only.

### 2.2. Screen-Printing Process

#### 2.2.1. Ink Types

The sensor was produced by screen printing functional inks directly on textiles. A UV-curable polymer ink from Electra Polymers Ltd. was used for the interface, dielectric layers, and encapsulation. A polymer silver ink was used to achieve the electrodes.

#### 2.2.2. Sensor Screen Printing

**Figure 6:**
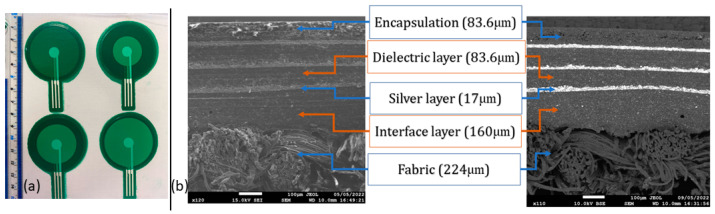
(**a**) The final printed sensor in four designs. (**b**) SEM images show the different layers and their corresponding average thickness (showing the thickness of each layer).

For this study, a white woven polyester/cotton fabric with a composition of 50% polyester and 50% cotton was selected as the substrate [25,26]. A 1-inch area of the chosen fabric was observed under a digital microscope and found to have a thread count of 4940, with an ends per inch (EPI) of 76 and a picks per inch (PPI) of 65. Additionally, polyester/cotton fabric is compositionally similar to the fabric used in hospitals’ personal protective equipment (PPE) and in patient gowns and is thus the most suitable for the intended application in this study [18]. This fabric was chosen because of its smooth surface, which was measured using a digital microscope with 3D scanning capability. A 0.1 cm radius circular area of the used fabric was examined under a digital microscope for surface roughness, which measured 119.24 μm. This roughness is likely to damage or cause discontinuities in the 10 μm thin silver ink-based electrodes. Therefore, the fabrication process begins with screen printing of a UV-curable dielectric ink/layer to act as an interface layer, which is cured in a UV conveyor for 20 s. The dielectric ink has a viscosity of 12,000 Cp and a dielectric constant of 3. This layer provides a smooth surface for the subsequent printing of the silver layer. The silver ink is ElectraPolymers Ronascreen 1300 Series Flexible Coverlay. As elaborated above, a 0.1 cm radius circular area of the interface layer was examined under a digital microscope and it was found that the surface roughness was reduced to 59.37 μm, although this is not as smooth as a standard polymer printing substrate, such as DuPont^TM^ Kapton^®^ [27], which has a surface roughness of 23 μm. High connectivity was observed with a 1.5Ω resistance between two distant points (distance = 3 cm) after depositing the silver layer which was cured in an oven at 110 °C for 10 min. The printing process involves repeatedly printing the dielectric layer and the conductive layer until all three electrodes and dielectric layers are completed. Figure 6a shows the printed sensors in four sensor designs made using this technique. Design 1 was examined using a scatter electron microscope to validate the continuity, positioning, and thickness of each layer, as shown in Figure 6b.

## 3. Results and Discussions

### 3.1. Characterisation of Screen-Printed Sensor

The permittivity values of various human body parts at 2.4 GHz, summarised in Table 1, were reported by Gabriel et al. in [28]. The body tissue was selected based on the expected position of the sensor in close proximity to the body. To mimic the permittivity values of human body fluids, deflated lungs, and muscles, three phantoms were created using water, acetone, and a customised gel mix, respectively. Table 1 provides a comparison between the permittivity values of human body tissues under investigation and lab-prepared mimicking phantoms. A glass jar with a wall thickness of 2.6 mm served as the container for the phantom. The purpose was to mimic the human body’s superficial subcutaneous adipose tissue in terms of permittivity and thickness.

**Figure 7:**
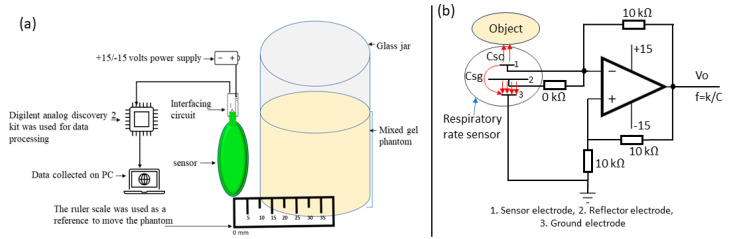
(**a**) Empirical setup to evaluate the sensor response toward phantoms. (**b**) The equivalent circuit model of the respiratory rate sensor and interfacing circuit.

The first empirical test was conducted to evaluate the sensor’s response to tissue-mimicking phantoms in close proximity. It is important to investigate the first 5 mm distance since the sensor would be directly printed on or embedded in the patient’s clothing, with proximity as close as 5 mm or less. For this initial testing, the measuring setup consists of a sensor plugged into an interfacing circuit with the help of standard card edge connectors, as shown in Figure 7a,b. The output of the interfacing circuit is connected through a BNC cable with channel-1 of the Digilent analog discovery-2, which is then connected to a computer. Initially, a glass jar filled with water was placed approximately 0.1 mm away from the sensor. Then, the water phantom was moved stepwise away from the sensor with 5 mm intervals, as shown in Figure 7a. Figure 7b shows the equivalent circuit model of the interfacing circuit and connected respiratory rate sensor, where the sensor and reflector electrodes are at the same phase and voltage. The reflector electrode directs the electric field of the sensor electrode toward the object. However, the fringing field between the sensor and the ground electrodes still exists (also shown in simulated work in Figure 4). This is minimised by selecting an optimal ratio of 1:3:1 for the sensor, reflector, and ground electrodes. The total capacitance ‘C_t_’ of the sensor is

C_t_ = C_sg_ + C_so_ (1)

In Equation (1), C_sg_ is the capacitance between the sensor and the ground electrode, and C_so_ is the capacitance between the sensor and the object that needs to be monitored. As elaborated above, the C_sg_ cannot be avoided due to the presence of a fringing field. However, it could be reduced to a minimum by selecting the appropriate ratios of electrodes. Furthermore, the oscillation frequency (f_0_) of the interfacing circuit in Figure 7b can be written as follows:

f_0_ = 1/2RC (2)

∆f/f_0_ = (∆C_t_)/(C_t0_ − ∆C_t_) (3)

f_0_ and C_t0_ represent the frequency and capacitance of the sensor in the absence of an object, while ∆f represents the corresponding change in frequency. When an object is brought closer to the sensor electrode, it causes a change in capacitance, which is represented as ∆C_t_ in Equation (3).

Table 2 provides the theoretical, simulated, and measured base capacitance values between the sensor and ground electrodes for design 2. Table 2 also shows a 1.875 pF reduction in the measured capacitance compared to the simulated capacitance. The lead capacitance cannot be avoided; however, its impact can be reduced. To minimise capacitance changes due to leads, the electronics were positioned in close proximity to the sensor, and the lead length was reduced to 20 mm with a thickness of 0.01 mm, as shown in Figure 6a. However, static capacitance from these shortened leads remains. The calculated capacitance between the leads is 0.00118 pF, whereas the calculated capacitance between the sensor electrode and ground electrode is 21.285 pF. Thus, it has been observed that reducing the lead length has mitigated its impact.

**Figure 8:**
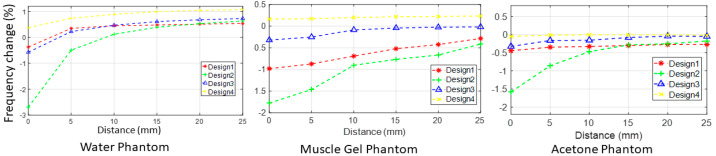
Frequency variation measurements obtained empirically when testing 3 different phantoms.

The percentage frequency change (%f-c) of four designs in response to the three different phantoms is presented in Figure 8. The %f-c due to the presence of three phantoms (water, acetone, and gel) at a distance of 0.1 mm can be summed up to obtain the cumulative %f-c, as listed in Table 3. According to Equation (4) and Figure 8, design 2 yields the maximum cumulative percent frequency changes of 6.2%f-c. In Equation (4), ‘D’ stands for design, ‘C’ stands for cumulative, ‘W’ represents water phantom, ‘G’ represents gel phantom, and ‘A’ represents acetone.

Σ D C %fc = %fc (W + G + A)(4)

Σ D2 C = (2.8 + 1.6 + 1.8) = 6.2%fc

**Figure 9:**
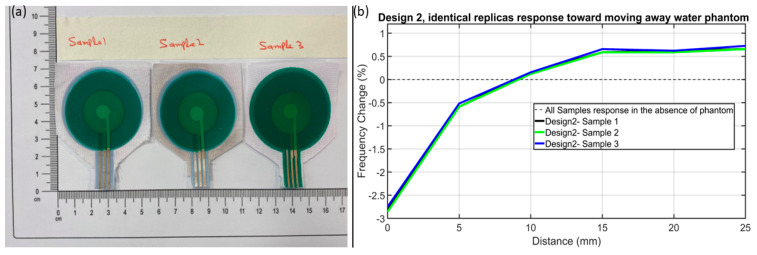
(**a**) Design 2’s identical replicas (Sample 1, 2, and 3) from three different batches of screen printing, and (**b**) their consistently similar %f-c toward mowing away water phantom.

Furthermore, the screen-printing reference marks help in repeatability and producing identical sensors of the same design, as shown in Figure 9a. Design 2’s three identical replicas from three different batches (shown in Figure 9a as Sample 1, Sample 2, and Sample 3) were selected for a repeatability test and their %f-c toward water phantom was tested. Figure 9b shows that identical sensors of the same design do produce a similar %f-c., which makes it evident that the proposed screen-printing mechanism and the sensor do have repeatability capability.

### 3.2. Environmental-Induced Drift

In a real-world scenario, encountering various forms of environmental-induced drifts is inevitable; in this research, these environmental-induced drifts will be referred to as noise. These environmental-induced drifts can originate from environmental fluctuations like changes in humidity and temperature, as well as motion artefacts such as flexing durability, pressure, and rubbing. Consequently, the performance of textile-based sensors can be affected, leading to potential inaccuracies and standard measurement uncertainties.

Graham reported in [29] that standard measurement uncertainties can be expressed as a standard deviation of the measurements from their base value in the presence of a specific noise (or induced drift).

To tackle this issue, an empirical detailed study was conducted to identify five primary sources of noise that can influence the sensor’s response and could introduce measurement uncertainties (or average standard deviations from their base values).

To ensure accurate sensor measurements, it is essential to determine if the cumulative effect of noise is outweighed by the sensor’s response to desired signals. The goal is for the sensor’s response to these phantoms to exceed the cumulative impact of all noise sources.

#### 3.2.1. Environmental Noise

Humidity and temperature variations are two environmental noises which could affect sensor response in a wearable scenario. To study the impact of these noises, the experimental setup elaborated in Figure 7a was repeated inside an environmental simulation chamber, which has the capability to keep one noise (either humidity or temperature) constant while changing the other one (either humidity or temperature).

#### 3.2.2. Humidity Impact

Humidity is essential for human comfort and well-being, but excessive levels can cause health problems. The ideal relative humidity percentage (RH%) range is 55% to 65%. High humidity can cause overheating and skin/eye irritation, while low humidity can worsen asthma and allergies [30].

**Figure 10:**
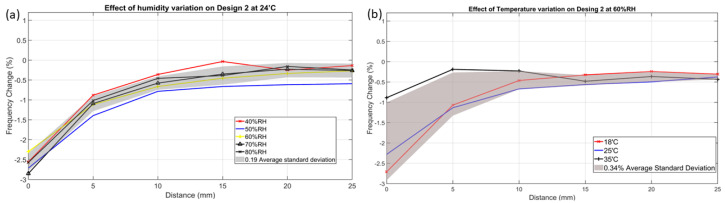
(**a**) Humidity variations ranging from 40% to 80% RH at 24 °C cause an average standard deviation of 0.19 in the design 2 response. (**b**) Temperature variations ranging from 18 °C to 35 °C at 60% relative humidity (RH) impact the response of design 2, resulting in an average standard deviation of 0.34.

The test was conducted to analyse and compare the impact of RH% variations on four designs. Figure 10a shows the response of design 2 to a water phantom’s movement at 24 °C with varying relative humidity from 40 to 80 RH%. The results indicate that a 40% humidity change significantly affects design 2, resulting in a 0.19 standard deviation of its %f-c. However, despite the significant impact of humidity variations, design 2 remains robust enough (6.2%f-c) to detect the dielectric changes as the phantom moved. The humidity impact on the rest of the three designs is given in Supplementary Data (S1).

The water phantom in a glass jar was utilised for this experiment, as depicted in Figure 7a. The fundamental working principle involves the sensor forming a capacitance with the water phantom through the glass wall of the jar. However, it has been observed that an increase in humidity levels leads to random water droplets forming on the outer side of the glass jar, due to condensation and causing dampness. Consequently, the sensor forms a capacitance with these random droplets as well as with water inside the glass jar. Therefore, this deviation results in the %f-c not following an increasing/decreasing pattern with increasing humidity. In addition, it has been noticed that humidity has a minimal effect on the sensor’s performance due to a 0.19 average standard deviation over all tested humidity levels.

#### 3.2.3. Temperature Impact

Temperature varies significantly throughout the year, ranging from 5 °C during winter to 40 °C in summer. To evaluate temperature’s impact on sensor behaviour and response, a controlled experiment varied temperature settings to 18 °C, 25 °C, and 35 °C while maintaining 60 RH% humidity. Figure 10b shows that the temperature variation immensely affected the sensor’s response. The impact of temperature on designs 1, 3, and 4 is provided in the supplementary data in S2. Additionally, when the temperature was raised to 45 °C, condensation occurred, dampening the sensor and electronics. As a result, the experiment was stopped before reaching 45 °C. Upon returning to room temperature, the sensor’s response returned to its original state, indicating the adverse effects of temperature were not permanent. This suggests that the sensor system is resilient and can adapt to varying temperature conditions.

### 3.3. Motion Artifacts

Since the sensor is designed to be worn by the patient, it is important to consider that it could be subject to flexing durability, compression, or rubbing caused by the movement of the human body and clothing movements.

#### 3.3.1. Flexing Durability Impact

**Figure 11:**
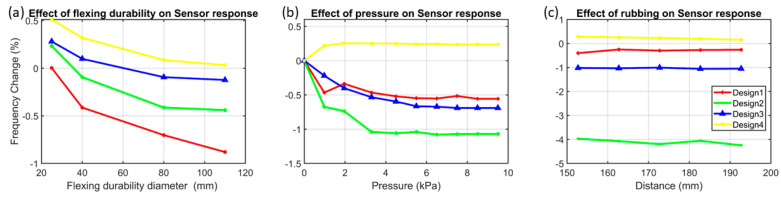
(**a**) The results of the flexing durability test conducted empirically on the four designs wrapped on cylinders of multiple diameters. (**b**) The graph shows the four designs’ responses to increasing pressure. (**c**) The graph shows the impact of rubbing on sensor response.

Flexing durability tests were conducted to assess and evaluate the sensor’s resilience to flexing durability. To perform the flexing durability test, the sensor was tapped on pipes of different sizes (with diameters ranging from 25 mm to 110 mm); the flexing durability setup is given in Supplementary Data (S3-a). To avoid pressure artefacts, the sensor was placed above the pipes. Although the test was performed with pipe diameters ranging from 25 mm to 110 mm, in a realistic scenario, it is highly unlikely for the sensor to be bent over a diameter of 25 mm. Therefore, the flexing durability region of interest is considered to be from 60 mm to 110 mm, the most likely flexing durability range. Design 1 exhibits the highest sensitivity (0.8%f-c) to flexing durability; it is not desirable because the ideal sensor would have minimal sensitivity to flexing durability. The flexing durability impact on each design is shown in Figure 11a and recorded in Table 4.

#### 3.3.2. Pressure Impact

In a real-time scenario, the sensor could undergo a change from 0.98 kPa (~10 g-force/cm^2^) to 10 kPa pressure (~101.9 g-force/cm^2^) [31]. Therefore, to conduct the pressure test, a plastic tube having lengths of 10 cm, 20 cm, and 30 cm was used, as shown in S3-b. Plastic tubes are utilised to prevent direct contact between the sensor and metal weights. This is because placing metallic weights on top of the sensor can increase its electrostatic capacitance, potentially affecting the pressure impact. Based on the recorded data from all four designs in Figure 11b, it is evident that design 2 is highly affected as it shows a maximum of 1.1%f-c with an applied pressure of 9.5 kPa (approximately 96 g-force/cm^2^). Each sensor response toward increasing pressure (~ or weight) was summarised in Table 4. Additionally, the sensor returns to its original 0% f-c after the applied pressure is removed. Furthermore, the sensor shows the same pattern of % f-c when the same pressure is applied cyclically.

#### 3.3.3. Rubbing Impact

Rubbing is another motion artefact which is most likely to occur in a real-time scenario when the patient has a sensor on their body. The rubbing experimental setup consists of the sensor, a fabric, and a glass beaker with added water, as shown in S3-c. The proposed sensor is taped on the table in such a way that the taping does not apply any pressure impact, except by holding the sensor in a fixed position. A fabric is then placed on top of the sensor, while the glass beaker with added water is placed on the fabric, as shown in S3-c. The fabric is slid over forward for 10 mm and then backward for 10 mm while recording the sensor’s response to each cycle. This combination of glass beaker with added water and sliding over on fabrics closely mimics real-time rubbing [28]. In some cases, the rubbing could be more intense; therefore, the water weight gradually increased from 10 g to 70 g. The glass beaker with water replicates a comparable property with human body fluids and fats, as elaborated in Section 3.1 [28]. Figure 11c shows the response of all four designs when subjected to rubbing, which results in less than 0.09%f-c on average, thereby demonstrating less influence from rubbing.

### 3.4. Comparisons of Noise and Sensor Response

The sensor’s reliability is determined by its ability to detect and differentiate phantoms, even in the presence of different environmental noise and motion artefacts. To assess this, different noises can be added up to find the cumulative impact in a linear manner, such as by calculating the following:

ΣC N = %fc (HV + FD + P + R)(5)

Σ D 2 C N = (0.19 + 0.61 + 1.1 + 0.09) %fc = 1.99%fc

In Equation (5), ‘N’ represents noise, ‘HV’ represents humidity variations, ‘FD’ represents flexing durability, ‘P’ represents pressure, and ‘R’ represents rubbing. The cumulative %f-c caused by noise at a temperature of 24 °C was determined using Equation (5) and recorded in Table 4. It can be observed that design 2 produces a 4.21% f-c output, despite being exposed to various forms of noise. This output is 2.4 times greater than the cumulative effect of the noise itself. Therefore, design 2’s response toward the dielectric phantom dominates the impact of all kinds of noise. It has been determined that design 2 is suitable for monitoring changes in phantoms’ dielectric properties that closely replicate human body movements. Furthermore, the following section will elaborate on the sensor’s reliability in respiratory rate monitoring of a test subject even in the presence of noise.

## 4. Respiratory Rate Monitoring

**Figure 12:**
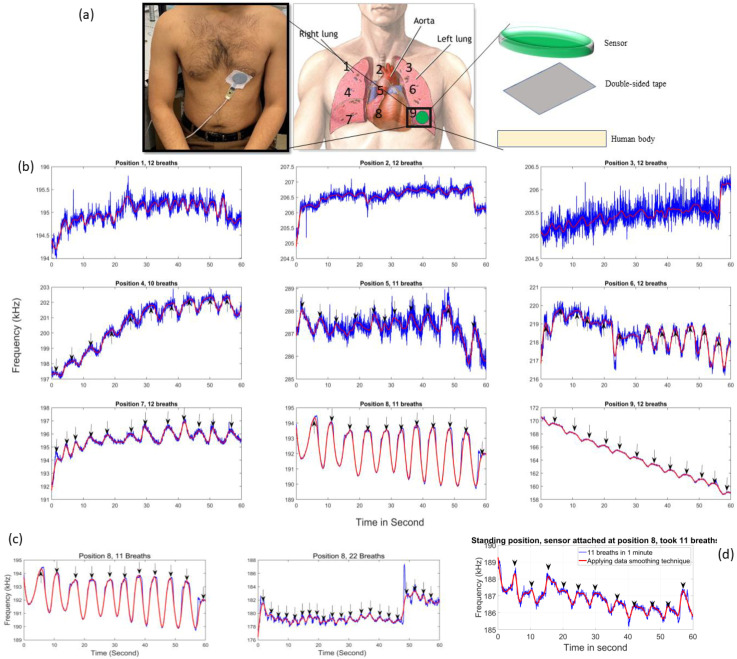
(**a**) The left image shows the sensor that is attached to the lower part of the chest of the test subject in a sedentary position. The middle image shows how the test subject’s torso was divided into nine positions. (**b**) The sensor was attached to each position and measured the corresponding breathing rate for one minute. Precise frequency peaks corresponding to the breathing rate can be seen when the sensor is attached at positions 4, 7, 8, and 9. (**c**) shows the sensor’s response for a random breathing rate of 11 and 22 in one minute. (**d**) The sensor is attached at position 8, while the test subject is in standing posture and took 11 breaths in one minute.

The working mechanism of the proposed sensor for respiration monitoring is based on recording the capacitance variations between the sensor electrode and lung inflation/deflation during inhalation and exhalation. One of the significant benefits of the proposed sensor is that it does not require any preparation of the patient’s body, such as shaving the area where the sensor is attached or tightly wrapping around the torso. This makes it more convenient for patients to wear the sensors throughout the day compared to the strain sensors reported in [1,11,21]. Although the proposed sensor is meant to monitor the respiratory rate of a test subject without direct skin contact, in this initial experiment, the sensor is attached to the test subject’s torso to analyse its accuracy and measure the maximum output in the form of frequency variation corresponding to inhalation and exhalation. The medical-grade double-sided tape received from 3M medical materials & technologies (3M United Kingdom PLC, Leicestershire, United Kingdom) was used to attach the sensor securely to the test subject’s torso, as shown in Figure 12a. The sensor wires were secured with medical-grade single-sided tape to prevent detachment during measurements. The test subject is a 31-year-old healthy male with a weight of 68 kg and a height of 171 cm, who did not have any chronic respiratory diseases or other active respiratory problems. To conduct the respiratory rate monitoring experiment, the test subject’s lung area was divided into nine mesh positions, as shown in Figure 12a. During this experiment, a 1 min time period was set on the stopwatch and the test subject was instructed to breathe continuously while manually counting their inhalations. The sensor response was initially measured at each position while the subject was sedentary, as depicted in Figure 12a,b.

To quantify the sensor response that was picked up during respiration, a minimum magnitude of 0.5 kHz was set between peaks and nulls to differentiate between inhaling and exhaling of breaths, as shown in S4 (Supplementary Data). A low-pass filter was applied (shown in red colour) on the raw data (shown in blue colour) to smooth the waveform. Positions 1, 2, and 3, which cover only a small part of the lungs, recorded muscle movements and body pressure instead of accurately capturing lung inflation and deflation during breathing. As a result, there is a lack of agreement between frequency peaks and the manually counted breaths, as shown in Figure 12b. Additionally, the frequency peak altitudes at these positions varied from 0.2 to 0.9 kHz. Due to the limited frequency variations between peaks and nulls, measurements at these positions are highly prone to environmental noise and motion artefacts.

The sensor detected ten peaks at position 4, which is in line with the subject’s breathing rate of ten breaths per minute (an arrowhead is also placed in the graphs to point out the counting peaks). This position covers the lungs, and the sensor is capable of accurately capturing lung expansion and contraction during breathing, resulting in precise nulls and peaks. Additionally, outlier data points were present in the raw data, but they were removed by applying a low-pass filter. At positions 5 and 6, the sensor detected 11 and 12 peaks, respectively, corresponding to inhaling 11 and 12 breaths per minute. However, the raw data signal contained numerous outlier data points, which were likely the result of heartbeats captured by the sensor, since the heart is also located at positions 5 and 6. The experiments continued at positions 7, 8, and 9 of the test subject’s torso, as depicted in Figure 12a.

The sensor detected 12, 11, and 12 precise peaks at positions 7, 8, and 9, respectively, which matched with the subject’s breathing rate of 12, 11, and 12 breaths per minute, as shown in Figure 12b. In addition, the sensor detected frequency peaks of 2.2, 3.7, and 1.3 kHz at positions 7, 8, and 9, respectively. Higher frequency peaks are preferred because they are less likely to be affected by human motion artefacts and white noise. It has been observed in Figure 12b–d that the experiments do not follow a single frequency. The designed sensor principle is to detect frequency variations (alternatively, capacitance variations) associated with breathing, rather than a single frequency, making the baseline frequency irrelevant.

The discrepancy in baseline frequencies at different measuring positions is attributed to variations in the surface area to which each position is attached, as well as differences in body tissues and bones at each position. Additionally, small ripples have been noticed in the sensor-recorded data, which is under investigation by increasing SNR and using signal-improving techniques such as signal estimation technique and probability theory, etc., to minimise the ripples’ impact while detecting peaks for the corresponding breath.

In order to test the accuracy of the sensor in measuring different breathing rates, the test subject was instructed to breathe at rates of 11 and 22 breaths per minute. The sensor was attached to position 8 on the subject’s torso, which had proven to be the most effective location in previous experiments. In this experiment, the sensor recorded 11 peaks for 11 breaths, and 20 peaks for 22 breaths (missing 2 breaths), as shown in Figure 12c. The recorded data also show that as the breathing rate increased, the sensor picked up frequency magnitude differences between peaks and nulls that were also shallowed. To test the sensor response toward their standing posture, the subject was instructed to stand while the sensor was still attached to position 8. Then, the test subject was instructed to breathe normally without having arm or foot movements. During this experiment, the test subject took 11 breaths in one minute. The sensor-recorded data are shown in Figure 12d, which gives 11 distinct peaks. However, in the standing posture, a higher amount of noise floor (ripples) can be seen; however, the peaks and nulls (corresponding to exhaling and inhaling) are still clearly identifiable. Additionally, when the test subject breathes in sitting postures, the abdomen (subsequently affecting lungs) is expanded more in the forward direction and less in the downward direction and vice versa in standing postures [32,33]. As the sensor is located in one position, the forward motion of the abdomen (alternatively, lungs) is more visible to the sensor, so the sensor recorded a clear peak and nulls at position 8 when the test subject is in the sitting position. On the contrary, in the standing position, the abdomen (alternatively, lungs) is less expanded forward and more expanded downward, so the sensor-recorded data in Figure 12d show some ripples in the raw data (in blue colour). Based on the experimental analysis, the proposed sensor reliably records respiratory rate in sedentary, standing positions and at a higher breathing rate even in the presence of environmental noise and motion artefacts. After conducting ten tests with different breathing rates, the proposed sensor has shown a high level of accuracy in measuring the respiratory rate, elaborated on in the Supplementary Data. It achieved an accuracy rate of 98.68%, indicating that the sensor can reliably capture and record the respiratory rate with precision (Supplementary Data elaborate on how the accuracy is calculated). The accuracy is calculated using a single test subject, where the proposed sensor is attached at different positions on the torso, and more than 10 RR tests are performed, while testing on different test subjects using the initial version of the proposed sensor is previously reported by the group in [23]. According to Table 5, the proposed respiratory rate sensor has advantages over other reported sensors in the literature due to ease of attachment, high immunity to environmental and motion noise, and last but not least, its high accuracy of 98.68%.

## 5. Conclusions

A respiratory rate sensor was optimised using different electrode ratios (1:1:5, 1:3:1, 1:4:2, and 1:5:5). The best designs were printed on fabric and analysed under a scanning electron microscope. Three phantoms, using acetone, water, and gel, were developed to mimic human body properties. Sensor design 2 outperformed others in response to phantom movement, producing a 6.2% frequency change (%f-c). Design 2 was robust enough to provide a 4.21%f-c in the presence of environmental noise, 2.4 times greater than all kinds of noise. Design 2 is highly sensitive to phantom movement, environmental noise, and motion artefacts. It has been chosen as the most accurate sensor for measuring respiratory rate and has been attached to a healthy male test subject at different positions on the torso. The results show precise measurements at positions 4, 7, 8, and 9. The sensor’s response at position 8 is particularly precise. It can measure respiratory rate accurately even when the test subject is breathing randomly or standing. More than ten RR tests on positions 4, 7, 8, and 9 were performed, which shows 98.68% accuracy. A small-sized microcontroller with wireless data transmission and a 3-volt battery is in the development phase to enable clinicians to access it remotely.

## Figures and Tables

**Table 1:** The dielectric properties of tissue types around the lung area at 2.4 GHz [28], and the materials used to build a human tissue empirical phantom model.

Tissue	Permittivity	Phantom Material	Permittivity
Fat	5.28	Glass	5
Deflated lung	20.5	Acetone	20.4
Inflated lung	48.4	Lab-prepared gel mix	50.6
Body fluid	78.2	DI water	80

**Table 2:** The base capacitance value comparison between theoretical, simulated, and measured sensor to ground electrodes.

Theoretical	Simulated	Measured
21.285 pF	21.675 pF	19.8 pF

**Table 3:** Sensor response toward water, acetone, and gel phantoms.

Design	Water	Acetone	Gel	Cumulative %f-c
	%f-c	%f-c	%f-c	
1	0.4	1	0.5	1.9
2	2.8	1.8	1.6	6.2
3	0.6	0.4	0.4	1.4
4	0.4	0.2	0.1	0.7

**Table 4:** The impact of various noises on sensor response in the form of %f-c due to the following parameters.

Design #	Environmental Noise	Motion Artefact			Cumulative %f-c due to All Noises	Cumulative %f-c with Phantoms (Given in Table 3)	Net %f-c
	Humidity	Flexing Durability	Pressure	Rubbing			
1	0.129	0.8	0.5	0.07	1.5	1.9	0.4
2	0.19	0.61	1.1	0.09	1.99	6.2	4.21
3	0.129	0.4	0.658	0.04	1.26	1.4	0.14
4	0.25	0.5	0.25	0.04	0.92	0.7	−0.22

**Table 5:** The proposed respiratory rate sensor comparison with the to-date reported respiratory rate sensors.

Ref.	PT a	Sensor Mounting Position	Monitoring Parameter	RRM b	Sensing Mechanism	IEMN c	MC d
[7,8]	No	Mouthpiece	Airflow	Yes	Resistive based	Not given	Not given
[9]	No	Mounted on torso	Chest vibrations	Yes	Resistive based	Not given	Not given
[13,14]	No	Mounted on torso	Chest applied pressure	Yes	Resistive based	Not given	Not given
[11]	No	Wrap around chest	Chest expansion	Yes	Strain based	Not given	Not given
[20]	No	Distant monitoring	Thorax movement	Yes	Capacitive based	Not given	Not given
[21]	No	Wrap around chest	Thorax movement	Yes	Capacitive based	Not given	Not given
This work	Yes	Anywhere on torso	Lung inflation and deflation	Yes	Capacitive sensing (capaciflector)	Sensor response 2.4-fold > sum of all noise	98.68%

a: printable on textile; b: respiratory rate monitoring; c: impact of environmental and motion noise; d: measurement accuracy.

## Data Availability

Data generated or analysed during this study are included in this article and in the Supplementary Data.

## Supplementary Materials

The following supporting information can be downloaded at: https://www.mdpi.com/article/10.3390/s24206513/s1.
