# Supplementary material for: A Novel Wearable Sensor for Measuring Respiration Continuously and in Real Time
Source: Sensors (Basel). 2024 Oct 10;24(20):6513. doi: 10.3390/s24206513 (PMC11511516; doi:10.3390/s24206513)
Supplement: Supplementary file 1 [file sensors-24-06513-s001.zip › sensors-3160701-supplementary.pdf]

Supplementary data of

A Novel Wearable Sensor for Measuring Respiration Continuously and in Real Time

Amjad Ali [1], Yang Wei [1,*], Yomna Elsaboni [1], Jack Tyson [2], Harry Akerman [3], Alexander I. R. Jackson [3], Rod Lane [4], Daniel Spencer [2] and Neil M. White [2]

[1] Smart Wearable Research Group, School of Science and Technology, Nottingham Trent University, Nottingham NG11 8NS, UK; amjad.ali@ntu.ac.uk (A.A.)
[2] School of Electronics & Computer Science, University of Southampton, Southampton SO17 1BJ, UK; nmw@ecs.soton.ac.uk (N.M.W.)
[3] Clinical Care, University Hospital Southampton NHS Foundation Trust, Southampton SO16 6YD, UK
[4] Zelemiq Ltd., Salisbury SP5 1EZ, UK; rod@zelemiq.com
* Correspondence: yang.wei@ntu.ac.uk; Tel.: +44-115-84-83372

Section 3-B-2

The average standard deviation calculation consists of calculating the standard deviation of the percentage frequency change at 0, 5, 10, 15, 20, and 25mm, which is due to the relative humidity change ranging from 40% to 80%RH. Then, the average of all standard deviations was calculated, as shown in the table below. The data below belong to design 2.

Table S1. Standard deviations (STD) calculations at each distance with varying relative humidity levels.

	**0 mm**	**5mm**	**10mm**	**15mm**	**20mm**	**25mm**	
Design_2_40%RH	-2.55577	-0.88106	-0.36011	-0.03496	-0.25173	-0.13635	
Design_2_50%RH	-2.71269	-1.3947	-0.78452	-0.66248	-0.61715	-0.59275	
Design_2_60%RH	-2.29668	-1.11693	-0.66318	-0.45375	-0.33508	-0.26876	
Design_2_70%RH	-2.85015	-1.0911	-0.57702	-0.34971	-0.22731	-0.26228	
Design_2_80%RH	-2.57223	-1.0148	-0.45807	-0.3876	-0.15856	-0.24665	
	STD	STD	STD	STD	STD	STD	Average STD
	0.206138	0.188685	0.166823	0.226515	0.178761	0.171549	0.189745

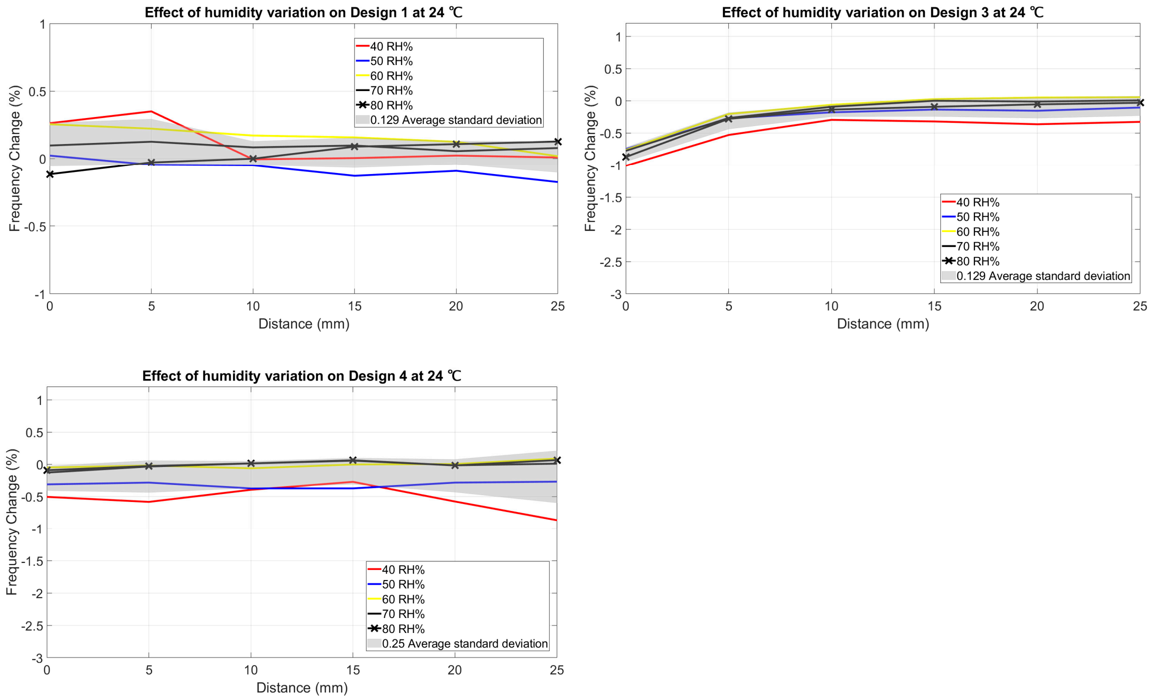

Figure S1. The impact of humidity variations from 40, 50,60,70 and 80 RH% at 24 °C, which gives a 0.129, 0.129, and 0.25 average standard deviation is shown by designs 1,3 and 4, respectively.

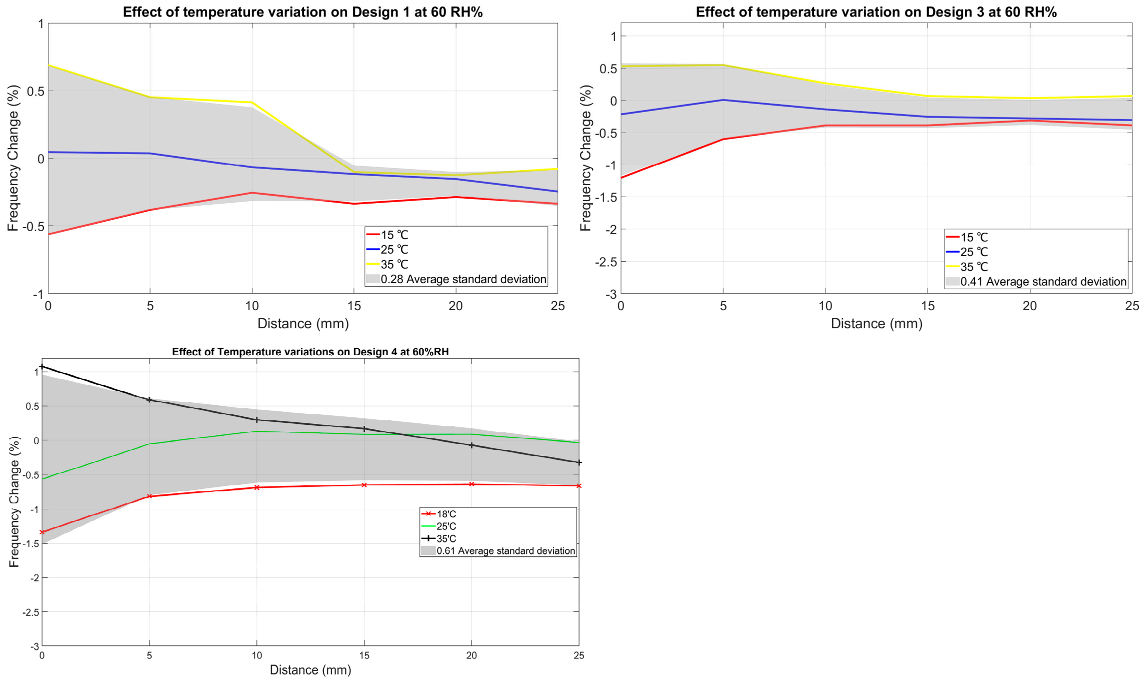

Figure S2. The impact of temperature variations from 18℃, 25℃, and 35℃ at a 60RH%, which gives 0.28, 0.41, and 0.61 average standard deviations is shown by designs 1,3 and 4, respectively.

Section 3-C

Empirical setup of motion artifacts such as bending, pressure, and rubbing.

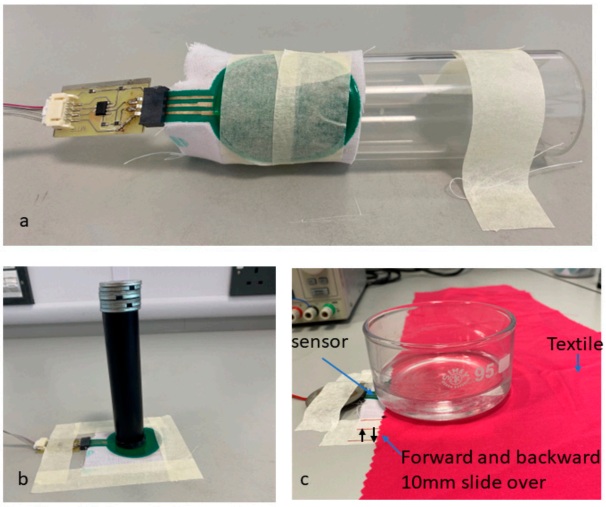

Figure S3. Empirical Setup used to evaluate the resilience of the designs to: (a) the flexibility durability where the sensor is tightly wrapped around 25, 40, 80, and 110 mm diameter cylinder and recorded its impact on sensor response; (b) the applied pressure and recording sensor response toward gradually increase of pressure (in the form of weight); (c) the rubbing, where the cloth is dragged back and forth for a distance of 10mm.

Section 4

Sensor accuracy toward respiratory rate

The accuracy of a sensor is an important factor in determining how well it responds to changing parameters it is meant to monitor. In the case of the proposed respiratory sensor, there are two ways to evaluate how accurately the sensor can monitor the respiratory rate of a test subject. The first approach involves testing the sensor response across multiple test subjects, and the second method is to test the sensor response at a different torso position and different breathing rate of a single test subject. We used the second method and tested the sensor response at a different torso position of a single test subject.

To quantify the sensor response which picked up during respiration, a minimum magnitude of 0.5 kHz was set between peaks and nulls to differentiate between inhaling and exhaling of breaths, as shown in Figure S-4. This technique of associating a peak with inhaling breaths was applied when the sensor was attached at positions 4 to 8, during breathing at varying rates of 10, 20, and 40, and in standing posture. The inhaling rate and corresponding peaks picked up by the sensor are given in Table 1. After conducting ten tests with different breathing rates, the proposed sensor has shown a high level of accuracy in measuring the respiratory rate. It achieved an accuracy rate of 98.68%, indicating that the sensor can reliably capture and record the respiratory rate with precision. However, the sensor response in positions 1, 2, and 3 was not considered in the accuracy calculation, as these areas contain a small part of the lungs.

Sensor accuracy calculations= (Number peaks picked up by sensor for below 10 experiments/ total counted manually breaths of the 10 experiments) *100

Sensor accuracy calculations= (149/151) *100=98.68

Table S1. Breaths taken by the test subject, and corresponding peaks picked up by the sensor.

Sensor attached position	Taken breaths by test subject	Peaks picked up by sensor	Incorrect picked up peaks by sensor
Position 4	10	10	0
Position 5	11	12	0
Position 6	12	12	0
Position 7	12	12	0
Position 8	11	11	0

Position 9	12	12	0
Position 8 at a rate of 11 breaths	11	11	0
Position 8 at a rate of 22 breaths	22	20	-2
Position 8 at a rate of 39 breaths	39	38	-1
Position 8 breathing in standing posture	11	11	0

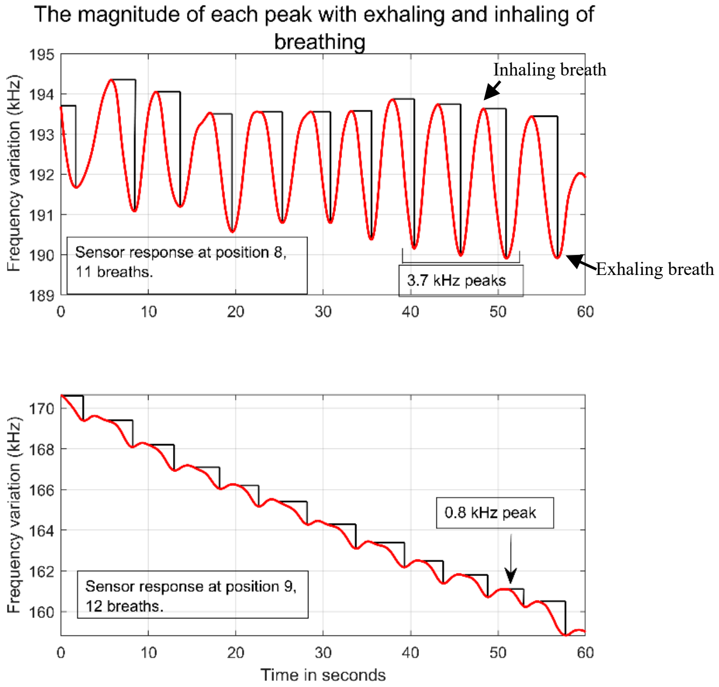

Figure S4. The sensor response at positions 8 and 9 shows a maximum of 3.7 and a minimum of 0.8 kHz peaks identified with inhaling the breaths, respectively.
